# Case report of Chagas disease reactivation: new diagnosis tool by direct microscopic observation of biopsy specimen and its preservation fluid

**DOI:** 10.1590/0037-8682-0326-2020

**Published:** 2020-12-21

**Authors:** Constanza Lopez-Albizu, Martín Pablo Bravo, Marisa Pico, Marisa L. Fernandez

**Affiliations:** 1 Instituto Nacional de Parasitología Dr. Mario Fatala Chaben, ANLIS Dr. Carlos G. Malbran, Departamento de Diagnóstico, Ciudad Autónoma de Buenos Aires, Argentina.; 2 Hospital Naval Dr. Pedro Mallo, División Infectología, Ciudad Autónoma de Buenos Aires, Argentina.; 3 Instituto Nacional de Parasitología Dr. Mario Fatala Chaben, ANLIS Dr. Carlos G. Malbran, Departamento de Clínica, Patología y Tratamiento, Ciudad Autónoma de Buenos Aires, Argentina.; 4 CEMPRA MT Hospital de Infecciosas Dr. F. J. Muñiz, Ciudad Autónoma de Buenos Aires, Argentina.

**Keywords:** Chagas disease reactivation, Meningoencephalitis diagnosis, Brain biopsy

## Abstract

Chagas Disease is caused by *Trypanosoma cruzi*. This infection is endemic in the Americas region. Neurological Chagas reactivation is diagnosed through the visualization of the parasite in the cerebrospinal fluid, blood, or tissue samples. Herein, we report the visualization of trypomastigotes by direct microscopic observation of a brain biopsy specimen and its preservation fluid (PF) in a paitient infected with VIH and *T. cruzi*. This easy and simple diagnostic method coupled with quantitative polymerase chain reaction can be used in all tissue biopsies and PF of *T. cruzi* seropositive patients, suspected of Chagas disease reactivation.

## INTRODUCTION

Chagas disease is a systemic disease caused by *Trypanosoma cruzi*. *T. cruzi* infection is endemic in the Americas, with about six million infected people in this region. However, this infection can be found worldwide due to migratory flow. *T. cruzi* is most commonly transmitted by vectors (kissing bugs), usually found in rural areas, and the transplacental route. Other possible transmission mechanisms include organ transplantation from an infected donor and transfusion of infected blood. Rarer transmission mechanisms include oral routes, sharing intravenous needles with an infected person, and laboratory accidents. Chagas disease is a neglected tropical disease that mostly affects people with poor socioeconomic status and those facing barriers for diagnosis, treatment, and control^1^.

Chagas disease reactivation is associated with immunodeficiency disorders, such as hematological malignancies, solid organ transplantations, or AIDS, and exhibits a high mortality rate in AIDS patients. It presents with neurological compromise in 75-90% of patients and acute myocarditis in 30% of patients. Other uncommon presentations include skin lesions, peritonitis, pleural effusion, gastrointestinal involvement, and cervicitis^2^ ^,^ ^3^. In neurological reactivation, patients usually develop fever, headache, seizures, intracranial hypertension, focal neurologic deficits, and progressive loss of consciousness. Neuroimaging scans reveal abnormalities in most patients, with brain masses observed in up to 85% of cases with compromised central nervous system (CNS). Single or multiple cerebral lesions are generally located in the subcortical white matter hemispheres, similar to toxoplasmosis infection findings^2^ ^,^ ^4^.

Acute neurological reactivation of chronic Chagas disease was first reported in 1969 in a patient with chronic lymphocytic leukemia^4^. The observation of trypomastigotes (the infective stage of the parasite) in the cerebrospinal fluid (CSF) and amastigotes (the replicative stage of the parasite) nests in the brain biopsy results of patients with HIV/AIDS was published in 1989 and 1990^5^ ^,^ ^6^. Neurologic Chagas reactivation diagnosis is based on the detection of trypomastigotes in CSF or blood or amastigote nests in tissue biopsies. Usually, trypomastigotes are found easily in the CSF when the CNS is compromised. They can also be found in peripheral blood using the Strout concentration method^2^ ^-^ ^4^ ^,^ ^7^. In one report, diagnosis was made by brain homogenate biopsy culture, and epimastigotes were observed after 72 hours of culture incubation^8^. Quantitative polymerase chain reaction (qPCR) for *T. cruzi* can be used in blood, CSF, or tissue analysis, but at the moment, this technique does not yield enough data to differentiate between chronic and acute infections. Therefore, qPCR is not considered for confirming reactivation.

In this case, we report the visualization of trypomastigotes by direct microscopic observation in a brain biopsy specimen and its preservation fluid (PF) taken from a patient infected with HIV and *T. cruzi*. To our knowledge, this diagnosis method has not been described in the literature for Chagas disease reactivation.

## CASE REPORT

**FIGURE 1:**
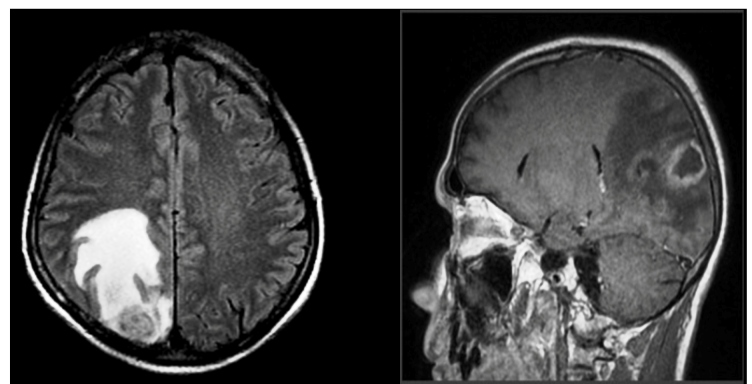
Brain magnetic resonance imaging of the patient shows a right parieto-occipital cortico-subcortical lesion with central necrosis areas and peripherally enhanced area surrounded by perilesional hyperintensity in fluid-attenuated inversion recovery and T2-weighted imaging.

A 46-year-old man born in the Tucuman Province, Argentina, with diagnosis of HIV infection and reactive serology for *T. cruzi*, was evaluated due to hemiparesis. Neuroimaging showed a cerebral mass (Figure 1). Lumbar puncture procedure to obtain CSF was contraindicated. Our institute was consulted for the first parasitemia by the Strout method, and the result was negative; qPCR *T. cruzi* DNA amplification was detectable in the peripheral blood with 14 parasite equivalents per milliliter of blood (par. Eq/mL)^9^. As previously mentioned, this result was not sufficient to confirm a reactivation diagnosis. Eight days later, brain stereotactic biopsy and a second parasitemia using the Strout method were performed. Chagas disease reactivation was finally diagnosed with the visualization of trypomastigotes using both the Strout method in peripheral blood and direct microscopy of the brain biopsy specimen and post-centrifuged pellet of PF. After diagnosis, the patient was treated with benznidazole and had a good clinical course.

The diagnosis of reactivation in the brain biopsy was quick and easy. A fragment of the biopsy specimen was placed on a microscope slide, covered with a cover glass, and observed directly with an optic microscope at 400× magnification. The microscopic observation revealed mobile trypomastigotes, some free in the liquid and others lodged in the brain tissue, both presenting the characteristic motility of *T. cruzi* trypomastigotes.

The procedure with the PF was performed by collecting fluid in a conical tube and centrifuging for 10 minutes at 3000 rpm. A fresh drop of the pellet was then obtained and observed at 400× magnification with an optic microscope. A significant number of typical flagellated trypomastigotes, which mobilized very quickly, were detected. The samples were tested for *T. cruzi* DNA using qPCR. A biopsy section was incubated in 200 μL of tissue lysis buffer (High pure template preparation kit, Roche Diagnostics GmbH, Mannheim, Germany). The DNA was eluted with 100 μL of elution buffer, according to the manufacturer’s instructions. Furthermore, DNA was obtained from 200 μL of the PF and eluted with 100 μL of elution buffer, according to the manufacturer’s instructions. The cycle threshold (Ct) of the brain biopsy and PF was 14.12 and 13.5, respectively. 

To estimate a parasitological imput on these samples, a *T. cruzi* DNA curve was made starting of distilled water spiked with culture of CL Brenner strain epimastigotes to obtain a concentration of 1 x 10^5^ epimastigotes/mL. The DNA was serially diluted with the elution buffer to obtain the following DNA concentrations: 10, 10^2^,10^3^, 10^4^, and 10^5^. The qPCR *of T. cruzi* DNA satellite amplification was performed^9^. The Ct of the highest concentration of 10^5^ parasites/mL in distilled water was 17.40.

### Ethics Considerations

This case report was approved by the Bioethics Committee of The National Institute of Parasitology, Dr. Mario Fatala Chaben, on April 24, 2020. 

## DISCUSSION

Chagasic meningoencephalitis is the most common presentation of reactivation in HIV-positive patients. CNS compromise is usually confirmed by the microscopic identification of trypomastigotes in CSF, positive Strout method result, or histopathological analysis of brain biopsies. In some cases, it is not possible to obtain CSF because Chagasic meningoencephalitis could produce large brain masses that are often contraindicated for a lumbar puncture, as in this case. The Strout method does not require any advanced technological procedures, only expert technicians.; however, its sensitivity is only 61.8%^7^. Even though Chagas Disease reactivation was highly suspected, it was not possible to diagnosed in the first Strout method performed. Histopathological diagnosis requires an invasive procedure, such as a brain biopsy, and the result may take a few days because of the number of steps (i.e., fixation, cutting, staining) required for observation. Molecular biology techniques, such as qPCR, are promising methods to detect Chagas disease reactivation; however, at the moment they do not have the capacity to differentiate between acute (including reactivation) and chronic infections in peripheral blood samples. There is limited information for using these techniques conclusively in samples such as CSF and tissues. It is known that in peripheral blood, *T. cruzi* DNA is detectable in approximately 50% of chronic patients without any immunosuppression^10^ ^,^ ^11^. Parasitic load analysis would be an early reactivation predictor, but currently, there is no cut-off value for the parasitic load to be conclusive. In the first samples of this case, qPCR in peripheral blood was detectable for *T. cruzi*, but the Strout method was negative. This could be explained by the parasitic load below the Strout method’s sensitivity threshold. This parasitic load, with our empirical experience, is not suggestive of an acute *T. cruzi* infection. As mentioned before, there is no parasitic load cut-off value to differentiate between acute or chronic infections. Coupling qPCR with brain biopsy could be another useful technique for parasite detection. It is important to consider the qPCR results. Presently, it takes at least 3 days to perform the test, the technique is not yet standardized for tissue samples, and there is no conclusive clinical trial on this method besides using peripheral blood for evaluation of trypanocidal treatment in chronic infection. In this case, the qPCR tests for brain biopsy and its PF showed low Ct values. Both values were under the minimum Ct obtained with 100.000 parasites/mL in the distilled water curve. This high burden of *T. cruzi* DNA detected by qPCR was consistent with the observed live trypomastigotes in both samples. 

Direct microscopic observation of brain biopsy and its PF can diagnose Chagas disease reactivation. The diagnosis can be made efficiently without any staining or other procedures, as we demonstrated that both samples were positive with the visualization of motile trypomastigotes. This diagnostic method can be useful in hospitals without the possibility to send the samples to reference laboratories where histopathological, qPCR, and cultures for *T. cruzi* are performed. 

If motile parasites are found in biopsied tissues or PF of an organ donor or organ explant, there is most likely a high parasitic load, which could mean a high parasitic replication rate. Thus, it could be a predictor of high-risk transmission or reactivation. Hence, we propose to implement this easy and simple diagnostic method with qPCR of all tissue biopsies and the PF of the biopsy or organs (i.e. donors tissues or organs) in *T. cruzi* seropositive patients.
